# Ketamine: A Neglected Therapy for Alzheimer Disease

**DOI:** 10.3389/fnagi.2019.00186

**Published:** 2019-07-24

**Authors:** Neil R. Smalheiser

**Affiliations:** Department of Psychiatry, Psychiatric Institute, University of Illinois School of Medicine, Chicago, IL, United States

**Keywords:** drug repurposing, discovery, literature based discovery, technological forecasting, neglected findings

## Introduction

Most attempts to identify promising new areas of scientific investigation rely on emerging trends such as seminal publications that become highly cited, or the appearance of increasing numbers of further articles on the same topic (Chen, 2006). Such bibliometric techniques follow research advances, but it would be valuable to predict trends before they have appeared in the published literature. Because of the uneven diffusion of research advances throughout science, a breakthrough which emerges in one domain area may take months or years before it becomes explored or applied in other fields. Thus, one can identify an emerging trend in one domain, and seek to predict new fields that might benefit. Some breakthroughs (e.g., the polymerase chain reaction) are clearly of wide applicability. However, some findings appear to have features or restrictions that are specific to one domain, which discourages investigators in another domain from exploring them. Such expectations create a delay or state of neglect in the second domain, even when a lack of information *per se* does not exist. In this paper, I will examine a breakthrough which arose in the field of depression research, which has not yet been explored in the context of another neuropsychiatric disease, namely, Alzheimer disease.

## Ketamine in Depression

One of the biggest breakthroughs in the study of depression has been the observation that ketamine, a NMDA antagonist drug used as an anesthetic and recreational drug, can reverse symptoms of depression within hours to days when given by intravenous infusion (Zanos and Gould, 2018). Ketamine has significant neurological and psychiatric side-effects, particularly when given chronically or at high doses, including dizziness, drowsiness, confusion, feelings of dissociation, insomnia, hallucinations, psychosis, and impaired learning and memory. Nonetheless, ketamine represents an exciting alternative to electroshock therapy in treatment-resistant depression, especially in patients with a high risk of suicide.

Several molecular effects of ketamine are linked to, and possibly underlie, its antidepressant actions:

- Activation of the mTOR pathway (Li et al., 2010). This is noteworthy since mTOR is a central controller of protein synthesis required for new synaptic connections (Gong et al., 2006).
- Phosphorylation of eukaryotic elongation factor 2 is inhibited, which augments subsequent expression of BDNF (Gideons et al., 2014).
- Although the proximal action of ketamine is upon NMDA receptors, secondary effects on AMPA receptors, metabotropic glutamate receptor mGlu2, and translocation of Gα_s_ from lipid rafts causing stimulation of cAMP appear to be critical for antidepressant efficacy (Gideons et al., 2014; Wray et al., 2018; Zanos et al., 2019b).

## Procognitive Effects of Ketamine in Human Depression and Animal Models of Depression

Activating mTOR, inhibiting Eef2 phosphorylation, and stimulating BDNF release should all stimulate synaptic plasticity and new memory formation within the brain. Indeed, low dose administration of ketamine exhibits procognitive effects, even when given for a prolonged period of time. In mice, chronic daily low dose (8 mg/kg) i.p. injections of ketamine avoided behavioral abnormalities such as disruption of sensorimotor gating, impaired cognitive function, and hyperlocomotor activity that were seen at higher doses (20 mg/kg) (Patrizi et al., 2015). In rats, 10 mg/kg produced sustained reversal of depression caused by chronic mild stress, with concomitant improvement of a cognitive task (novel object recognition task) (Papp et al., 2017). In another study, ketamine (10 mg/kg) preserved extra-dimensional (ED) set-shifting ability, a form of cognitive flexibility, which is impaired in rats subjected to repeated restraint stress (Nikiforuk and Popik, 2014). In humans, acute low dose sublingual ketamine helped visual memory, simple working memory, and complex working memory in individuals with treatment-resistant depression (Lara et al., 2013; Zhang and Ho, 2016). Lee et al. (2016) have proposed that ketamine's antidepressant actions may, in fact, be a consequence of its procognitive effects.

## Ketamine vs. Memantine in Alzheimer Disease

To my knowledge, only Lozupone et al. (2018) have explicitly discussed the possibility that ketamine may represent a potential therapy for Alzheimer disease, and they did so as part of a review of treatment options for depression co-occurring in Alzheimer patients. The lack of ongoing research about ketamine is not surprising, given that it can cause loss of new memories, confusion, dissociation, even psychosis—all of which are symptoms or worrying comorbidities of Alzheimer disease itself. No wonder that a search of Clinicaltrials.gov (carried out in April 2019) shows 152 trials of ketamine in depression, and 38 trials that involve various antidepressants in Alzheimer patients, but no registered trials that involve giving ketamine to Alzheimer patients.

On the other hand, memantine is one of the most prescribed and most investigated drugs employed in Alzheimer patients—and both ketamine and memantine are non-competitive NMDA receptor antagonists with similar, though non-identical, pharmacologic profiles. Compared to ketamine, memantine exhibits lower affinity, fast channel-blocking kinetics, and strong voltage-dependence (allowing rapid relief of block during synaptic activity), as well as reduced trapping (permitting egress from closed channels). These factors may allow memantine to block channel activity induced by low, tonic levels of glutamate–an action that might contribute to symptomatic improvement and could theoretically protect against weak excitotoxicity–while sparing synaptic responses required for normal behavioral functioning, cognition, and memory (Rogawski and Wenk, 2003). Memantine is only modestly effective in improving cognition and behavioral disturbances in Alzheimer patients (Kishi et al., 2017b; McShane et al., 2019), but it produces much fewer adverse side effects than ketamine at clinically prescribed doses.

Substantial overlap might be expected between the effects of ketamine and memantine, and it is difficult to assess how many of the reported differences are quantitative rather than truly qualitative in nature. Memantine exhibited antidepressant-like effects in some, but not all, animal models of depression (Zhang et al., 2017; Amidfar et al., 2018; Takahashi et al., 2018). However, in most studies, the molecular effects of ketamine which are associated with antidepressant activity are not produced by memantine (Gideons et al., 2014), and indeed, memantine has failed to produce rapid antidepressant effects in human clinical trials of depression (Kishi et al., 2017a). This suggests that ketamine's antidepressant (and procognitive) effects are not entirely shared with memantine.

## Avoiding the Side Effects of Ketamine

As stated above, low dose sublingual administration of ketamine appears to be safe, and might be tried as a procognitive therapy in Alzheimer patients instead of memantine. However, the risk/benefit considerations for treating Alzheimer patients chronically are quite different than for preventing suicide acutely in severely depressed patients. One needs to be convinced that the risk of side effects is truly minimal. In the context of depression, research continues actively on the best doses, duration, and methods of administration of ketamine, and there are several promising leads to avoid side effects. For example, (R)-ketamine exhibits fewer side effects than racemic ketamine or (S)-ketamine (Chang et al., 2019; Zanos et al., 2019a). A metabolite of ketamine, (S)-Norketamine, is reported to be effective as an antidepressant without eliciting the side effects observed with (S)-ketamine (Yang et al., 2018a). Perhaps most exciting of all, (2*R*,6*R*)-Hydroxynorketamine [(2*R*,6*R*)-HNK] elicits rapid antidepressant actions in rodents. Like ketamine itself, this metabolite increases the level of the AMPAR protein receptor (Zanos et al., 2016) and stimulates BDNF, mTOR, and cAMP (Zanos et al., 2016; Wray et al., 2018; Fukumoto et al., 2019). There is controversy whether all of the actions of ketamine are due to (2*R*,6*R*)-HNK, and regarding whether or not (2*R*,6*R*)-HNK binds NMDA receptors (Kavalali and Monteggia, 2018; Lumsden et al., 2019). However, the antidepressant effects of (2*R*,6*R*)-HNK were not accompanied by NMDA receptor-dependent side effects, suggesting that ketamine metabolites may provide a safer means for treating depressed patients—and by implication, a safer venue for testing procognitive effects in Alzheimer patients.

## Arrowsmith Two-Node Search

I carried out an Arrowsmith two node search (http://arrowsmith.psych.uic.edu/cgi-bin/arrowsmith_uic/start.cgi) to look for so-called B-terms (shared title words and phrases, representing concepts and molecules) that might link the ketamine vs. Alzheimer literatures in a meaningful way (Torvik and Smalheiser, 2007; Smalheiser et al., 2009). Four of these B-terms are worth mentioning here:

- **VGF**. VGF is downregulated in depressed human subjects and in mice subjected to chronic social defeat stress, and ketamine stimulates VGF translation (Jiang et al., 2018). This is noteworthy since VGF is significantly and robustly downregulated in CSF (Carrette et al., 2003) and postmortem brains (Cocco et al., 2010) of Alzheimer patients. If ketamine stimulates VGF in these patients, it may be addressing one of the molecular abnormalities contributing to AD.
- **mTOR (mTORC1)**. As mentioned above, ketamine activates mTOR. However, the literature suggests that mTOR is already upregulated in Alzheimer disease brains (Tramutola et al., 2015), animal models (Caccamo et al., 2018), and in Down Syndrome (Perluigi et al., 2014) whose patients exhibit accelerated AD pathogenesis. In fact, mTOR inhibition has been proposed as a means of slowing AD pathogenesis and brain aging in general (Richardson et al., 2015; Caccamo et al., 2018). This is a key point that needs to be considered in any exploration of ketamine. It is not clear whether ketamine would further activate mTOR in AD patients, or whether ketamine's antidepressant or procognitive actions would be abrogated in brains whose mTOR is upregulated. Possibly the combination of ketamine with additional agents [e.g., the mTORC1 inhibitor rapamycin, which on its own has memory-improving effects (Richardson et al., 2015)] would have protective or synergistic effects.On the other hand, one study of a chronic social defeat model of depression found that mTOR plays a role in the antidepressant effects of (S)-ketamine, but not the enantiomer (R)-ketamine, whereas ERK plays a role in (R)-ketamine's antidepressant effects (Yang et al., 2018b). In fact, acutely administered (R)-ketamine was reported to have rapid antidepressant-like effects but not to alter mTOR signaling (Yang et al., 2018a). This may favor (R)-ketamine as a preferred drug to study.
- **Autophagy**. Autophagy is a normal process of clearing dead cells and removing cellular organelles and other components; this process is generally thought to be deficient in AD brains and to contribute to the pathogenesis of the disease (Zare-Shahabadi et al., 2015). Autophagy in AD may be suppressed, at least in part, by the upregulation of mTOR just discussed (Zare-Shahabadi et al., 2015). Interestingly, one study in Wistar-Kyoto rats, a putative animal model of depression, found that chronic administration of ketamine for 11 days at very low doses (0.25 or 0.5 mg/kg) did elevate phosphorylated mTOR in the hippocampus, yet did not affect autophagy markers (Akinfiresoye and Tizabi, 2013). In another study of normal mice, chronic administration of ketamine for 3 weeks (5–10 mg/kg) did not inhibit autophagy markers (Kara et al., 2017).More research is clearly needed on the relationship of ketamine to mTOR and autophagy, but several studies suggest that choosing the right form of ketamine, the right dose, or a chronic paradigm may avoid not only the side effects on memory and cognition, but also the potentially undesirable molecular effects on mTOR and autophagy as well.
- **Inflammatory cytokines**. In models of depression in both mice and rats, the rapid antidepressant-like effects of ketamine (10–20 mg/kg given acutely) are associated with a decrease in pro-inflammatory cytokines in serum and brain tissues (Yang et al., 2013; Wang et al., 2015; Clarke et al., 2017; Tan et al., 2017; Xie et al., 2017). This is interesting in view of the emerging evidence that pro-inflammatory cytokines are elevated in Alzheimer disease and that neuroinflammation has a contributory role in AD pathogenesis (Heneka et al., 2015). Ketamine (1 or 7 mg/kg) also reduces lipopolysaccharide-induced inflammatory cytokines in serum (Ward et al., 2011). It should be cautioned that, to my knowledge, effects of chronic low dose ketamine on cytokines have not been examined in animals. Moreover, the role of inflammation is not straightforward in humans; for example, patients with AD exhibit an upregulation of both pro-inflammatory and anti-inflammatory cytokines in the CSF (Taipa et al., 2019). Nevertheless, documented effects of ketamine on inflammatory cytokines add to the rationale for its study in AD.

## Conclusion

The mechanisms by which ketamine ameliorates symptoms of depression are not fully known, and the optimal doses, forms, and routes of administration are not yet worked out. The application of knowledge from depression to Alzheimer disease is fraught with uncertainty. There are, indeed, valid concerns to explain why ketamine has not previously been explored in this context! Nevertheless, a review and analysis of the existing literature provides a reasonably strong scientific rationale to encourage testing whether ketamine (or its metabolites) have procognitive effects in Alzheimer patients.

## Author Contributions

NS designed, analyzed, and wrote the article.

### Conflict of Interest Statement

The author declares that the research was conducted in the absence of any commercial or financial relationships that could be construed as a potential conflict of interest.
